# Potential of Inulin-Fructooligosaccharides Extract Produced from Red Onion (*Allium cepa* var. *viviparum* (Metz) Mansf.) as an Alternative Prebiotic Product

**DOI:** 10.3390/plants10112401

**Published:** 2021-11-07

**Authors:** Jakkrit Aisara, Pairote Wongputtisin, Somkid Deejing, Chutamas Maneewong, Kridsada Unban, Chartchai Khanongnuch, Paul Kosma, Markus Blaukopf, Apinun Kanpiengjai

**Affiliations:** 1 Graduate Program in Biotechnology, The Graduate School, Chiang Mai University, Chiang Mai 50200, Thailand; jakkrit_ai@cmu.ac.th; 2 Division of Biochemistry and Biochemical Technology, Department of Chemistry, Faculty of Science, Chiang Mai University, Chiang Mai 50200, Thailand; 3 Program in Biotechnology, Faculty of Science, Maejo University, Chiang Mai 50290, Thailand; pairotewong@gmail.com (P.W.); kittydeejing@gmail.com (S.D.); chutamas_m@yahoo.com (C.M.); 4 Division of Biotechnology, School of Agro-Industry, Chiang Mai University, Chiang Mai 50100, Thailand; kridsada_u@cmu.ac.th (K.U.); chartchai.k@cmu.ac.th (C.K.); 5 Department of Chemistry, BOKU University of Natural Resources and Life Science, 1190 Vienna, Austria; paul.kosma@boku.ac.at (P.K.); markus.blaukopf@boku.ac.at (M.B.); 6 Research Center of Microbial Diversity and Sustainable Utilization, Faculty of Science, Chiang Mai University, Chiang Mai 50200, Thailand

**Keywords:** red onion, fructooligosaccharides, prebiotics, bifidogenic effect

## Abstract

Red onion is a popular ingredient in many Thai dishes and has recently been promoted for commercial cultivation. In this study, inulin-fructooligosaccharides (inulin-FOSs) were extracted from red onions in a simplified extraction method. The extract contained 24.00 ± 0.38 g/L free glucose, fructose and sucrose, while the level of FOSs was recorded at 74.0 ± 2.80 g/L with a degree of polymerization of 4.1. The extract was resistant to simulated gastrointestinal conditions, while selectively promoting probiotic lactobacilli. These outcomes resulted in inhibitory effects against various pathogenic bacteria. The in vitro batch culture fermentation of the extract by natural mixed culture indicated that an unknown sugar identified as neokestose was more rapidly fermented than 1-kestose and other longer-chain inulin-FOSs. Notably, neokestose selectively encouraged a bifidogenic effect, specifically in terms of the growth of *Bifidobacteirum breve*, which is an infant-type probiotic bacterium. This is the first report to state that neokestose could selectively enhance the bifidogenic effect. In summary, inulin-FOSs extract should be recognized as a multifunctional ingredient that can offer benefits in food and pharmaceutical applications.

## 1. Introduction

Approximately 80% of the dry matter of onion bulbs (*Allium cepa* L.) is composed of nonstructural carbohydrates that basically consist of glucose, fructose, sucrose and fructooligosaccharides (FOSs) [1]. After chicory root, *A. cepa* L. is recognized as the second most abundant source of FOSs. This has been structurally identified as inulin-type FOSs or inulin-FOSs [2]. Inulin-FOSs are produced and subsequently accumulated in onion bulbs, while their amounts and components vary depending upon the variety, leaf-base and quality of the onion bulb [1,3]. As far as this report is concerned, FOSs are one of the most acceptable prebiotic substances of oligosaccharide prebiotics; i.e., galactooligosacharides (GOSs) and lactulose [4]. 

Based on their color, onion bulbs are classified as yellow, red and white. They are also classified according to their sweetness as being either sweet or non-sweet [5]. White onion has a sweet taste, and neither the fresh nor cooked form is preferably consumed among Thai people. In contrast, yellow onions are less sweet than white onions and are more traditionally consumed in Thailand. Red onion (*Allium cepa* var. *viviparum* (Metz) Mansf.) or Hom-kaek looks like a shallot but is sweeter. Red onions are familiar to Thai people, and their commercial cultivation is currently being promoted. As stated in the findings of a previous study, the variety of onion species may provide a distinct pattern of FOSs [6] that consequently influences the prebiotic activities of the onion. 

In a previous study, inulin-FOSs were extracted from yellow and red onions in order to compare their inulin-FOSs profiles. It was found that the red onion is a potential source of inulin-FOSs in terms of quality and quantity [7]. Furthermore, the extracted inulin-FOSs are sweet and could be considered for use as a functional syrup. However, the deep-rooted functions of inulin-FOSs extract with regard to potential changes in microbiota have not yet been studied in terms of its potential health benefits [8]. Some distinct patterns of the inulin-FOSs extracted from red onions were identified in this study. It is expected that the suspected oligosaccharides may exhibit prebiotic properties and may promote more efficient prebiotic effects than the reported inulin-FOSs.

To be a prebiotic substance, a substance must be resistant to the digestive enzymes of the host and must be selectively fermented by the microorganisms present in the host’s intestinal tract [9,10]. The aims of this study were to evaluate the prebiotic properties of the inulin-FOSs extract. Accordingly, experiments were divided into two main phases. Firstly, we examined the digestibility of inulin-FOSs extract under simulated gastrointestinal tract (GIT) conditions. Secondly, we studied its fermentability towards various probiotic lactic acid bacteria and microbial community changes during fermentation by human normal flora derived from healthy infants as a potential model for further product development.

## 2. Results

### 2.1. Inulin-FOSs Extract Obtained from Red Onions

Inulin-FOSs were extracted from red onions using a simplified method. The resulting extract was considered inulin-FOSs syrup consisting of 98.00 ± 0.28 g/L total carbohydrates and included 6.95 ± 0.29 g/L glucose, 3.23 ± 0.24 g/L fructose and 13.76 ± 0.24 g/L sucrose, resulting in approximately 74.00 ± 2.8 g/L of inulin-FOSs with an average degree of polymerization (DP) of 4.1. Based on the fresh weight of the red onions, the total extract yielded 3.7 g inulin-FOSs per 100 g of fresh red onions (Table 1). 

### 2.2. Digestibility of Inulin-FOSs Extract under Simulated Gastrointestinal Tract (GIT) Conditions

**Figure 1:**
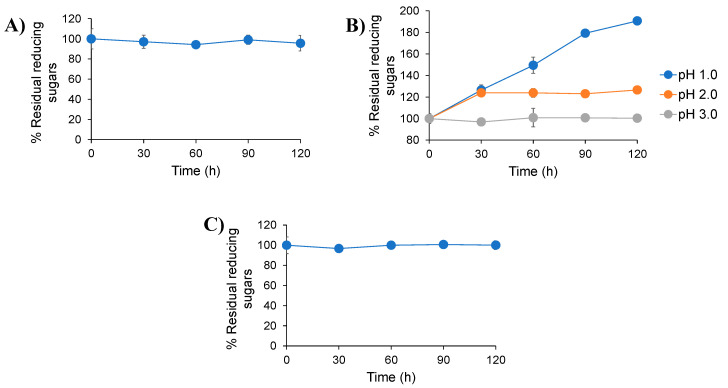
Residual reducing sugars retained after treatment of inulin-FOSs extract with simulated oral (**A**), gastric (**B**) and intestinal (**C**) conditions.

**Figure 2:**
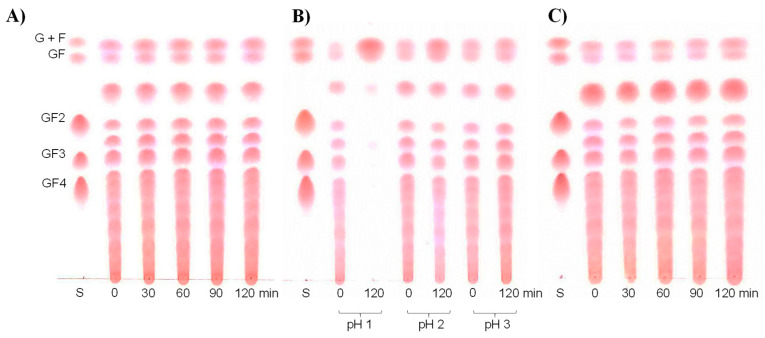
Thin-layer chromatography (TLC) chromatograms displaying profiles of residual sugars and oligosaccharides after treatment of inulin-FOSs extract under simulated oral (**A**), gastric (**B**) and intestinal (**C**) conditions. G = glucose; F = fructose; GF = sucrose; GF2 = kestose; GF3 = nystose; GF4 = 1-F fructofuranosylnystose.

GIT conditions, including oral, gastric and small intestinal conditions, were simulated based on the presence of salivary α-amylase, pepsin and acidity, along with a combination of different digestive enzymes and bile salts. The incubation step was carried out under conditions simulating the normal temperature of the human body at 37 °C. Experiments were carried out under appropriate conditions with regard to the transit time of food in the mouth, the stomach and the small intestines of humans. The results revealed that inulin-FOSs were not affected by salivary α-amylase and the combination of pancreatin and bile salts. This was established according to the fact that no differences were observed in the retained reducing sugars (Figure 1 and Figure 2). However, under gastric conditions, where the pH values were less than 2.0, the inulin-FOSs were partially digested to both mono- and disaccharides. 

### 2.3. Inulin-FOSs Extract Fermentation and Inhibitory Effect on Foodborne Pathogenic Bacteria

**Figure 3:**
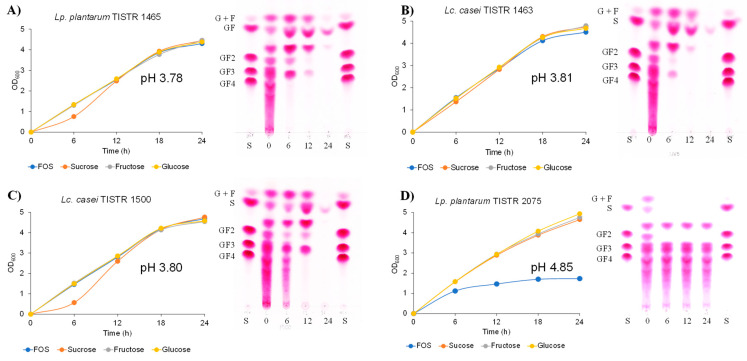
Growth and TLC chromatogram of different probiotic *Lc. casei* TISTR 1463 (**A**), *Lp. plantarum* TISTR 1465 (**B**), *Lc. casei* TISTR 1500 (**C**) and *Lp. plantarum* TISTR 2075 (**D**) when cultivated in inulin-FOSs extract at 37 °C for 24 h.

The inulin-FOSs extract was fermented with the use of four representative probiotic bacteria: *Lacticaseibacillus casei* TISTR 1463 [11], *Lactiplantibacillus plantarum* TISTR 1465, *Lc. casei* TISTR 1500 [12] and *Lp. plantarum* TISTR 2075 [13]. As was determined by the optical density of the fermentation broth at 600 nm (OD_600_), the first three types of probiotic lactic acid bacteria were able to almost completely ferment inulin-FOSs when compared with the other fermentable sugars that are typically found in red onions (glucose, fructose and sucrose) (Figure 3).

The latter probiotic lactic acid bacterium, *Lp. plantarum* TISTR 2075, could partly ferment inulin-FOSs as it yielded an approximately five-fold lower value of OD_600_ than those fermented by glucose, fructose and sucrose. This was due to the fact that its fermentation was found only in glucose, fructose, sucrose and 1-kestose. These results are in accordance with the relevant carbohydrate profiles analyzed by TLC and indicated a decrease in pH values at the end of the fermentation periods. The fermentation broth obtained from *Lc. casei* TISTR 1463, *Lp. plantarum* TISTR 1465 and *Lc. casei* TISTR 1500 was then tested for potential antimicrobial activities. Both the non-neutralized fraction and the neutralized fraction of the fermentation broths displayed apparent inhibitory effects against *Escherichai coli*, *Bacillus cereus*, *Salmonella enterotica* ser. Thyphimurium, *Staphylococcus aureus* and *Listeria monocytogenes*. However, the non-neutralized fraction displayed a significantly greater degree of inhibitory activity than the neutralized fraction (Table 2).

### 2.4. Effect of Inulin-FOSs Extract on Natural Mixed Culture

**Figure 4:**
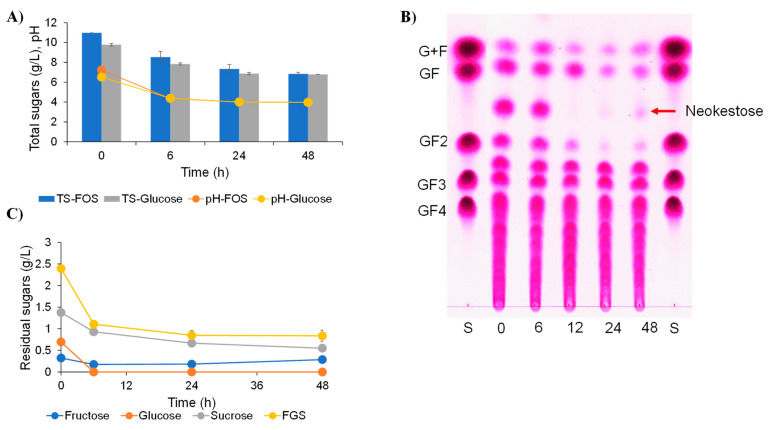
Profile of residual total sugars, pH values compared with the control (glucose) (**A**), TLC chromatogram (**B**) and residual fructose, glucose, sucrose and the sum of the residual sugars fructose, glucose and sucrose (FGS) (**C**) during cultivation of inulin-FOSs extract at 37 °C for 24 h.

**Figure 5:**
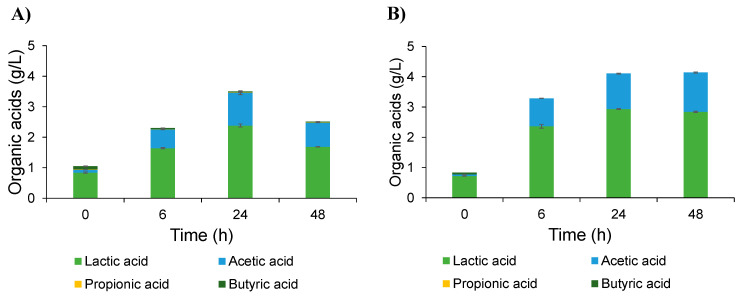
Lactic acid and SCFAs profiles of inulin-FOS extract fermentation (**A**) compared with those of glucose fermentation (**B**).

The fermentability of the inulin-FOSs was evaluated using a natural mixed culture obtained from the fecal slurry of a healthy infant. The glucose treatment was used as the control treatment. The results indicated that the natural mixed culture consumed inulin-FOSs in a manner similar to glucose. The total carbohydrate contents obtained from the two simulated fermentation conditions were dramatically decreased from 10 g/L to 6.5 g/L after 48 h of fermentation. This outcome was in accordance with the pH values, which initially declined from pH 6.8 to pH 4.0 at end of the fermentation process (Figure 4a). The TLC chromatogram revealed that glucose and/or fructose, 1-kestose and an unknown sugar were fermented, while other oligosaccharides had no effect on the fermentation process (Figure 4b). Glucose was completely consumed within 6 h of the fermentation process, while sucrose was partly used, and fructose remained constant throughout the process of fermentation (Figure 4c). Notably, the unknown sugar played a crucial role in the inulin-FOSs fermentation process as it was consumed within 12 h of fermentation. This occurred 6 h faster than for glucose consumption, but its structure must be further investigated and identified. The short-chain fatty acid (SCFA) profile revealed that lactic acid was the main organic acid initially obtained from the fecal slurry, while trace amounts of acetic acid and butyric acid were also observed. After the fermentation of inulin-FOSs and glucose, only lactic acid and acetic acid were predominantly produced; however, they were found to have been produced in quantities that significantly differed. The inulin-FOSs system seemed to induce the production of SCFAs rather than glucose (Figure 5). 

In total, 803,075 sequencing reads were obtained from a single run of 8 samples of the genomic DNA extracted from the cell pellets of microorganisms that were cultured by inulin-FOSs and glucose. Of these, low-quality and chimera sequences were removed. Consequently, 710,170 reads with an average length of approximately 417 nucleotides (nt) were obtained for further analyses. To classify the bacteria associated with fermentation, nucleotide sequences were clustered with 97% identity and expressed in terms of operational taxonomic units (OTUs). Overall, the OTUs ranged from 350 to 631 OTUs with an average of 416 OTUs and were identified according to phylum, family, genus and species.

**Figure 6:**
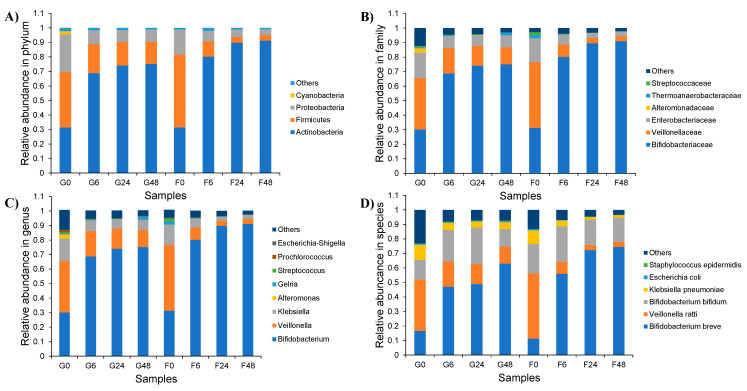
Relative abundance of bacterial community at the phylum (**A**), family (**B**), genus (**C**) and species (**D**) levels during inulin-FOSs extract and glucose fermentation.

**Figure 7:**
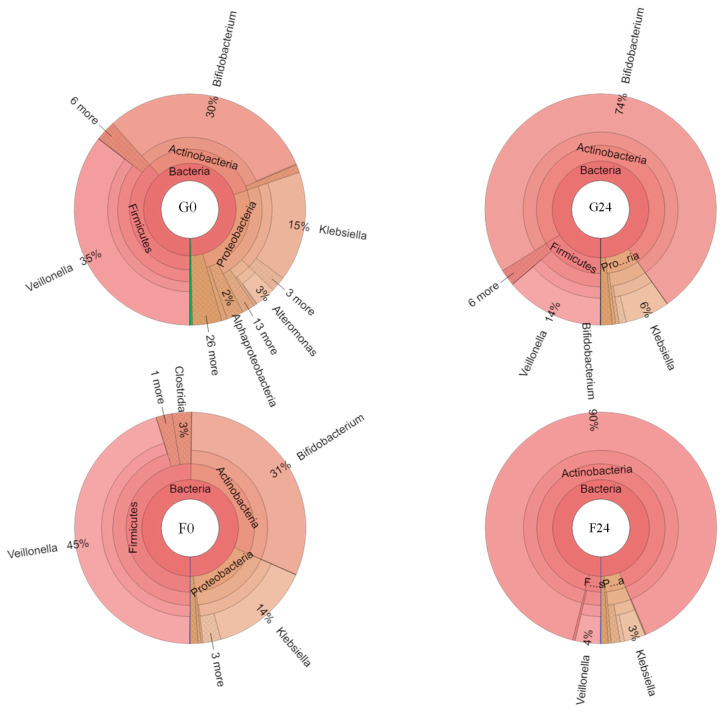
Krona graphs based on relative abundance of OTU up to the bacterial genus level obtained from the fermentation of glucose (G0, G24) and inulin-FOSs extract (F0, F24) at 0 and 24 h of the fermentation processes.

**Figure 8:**
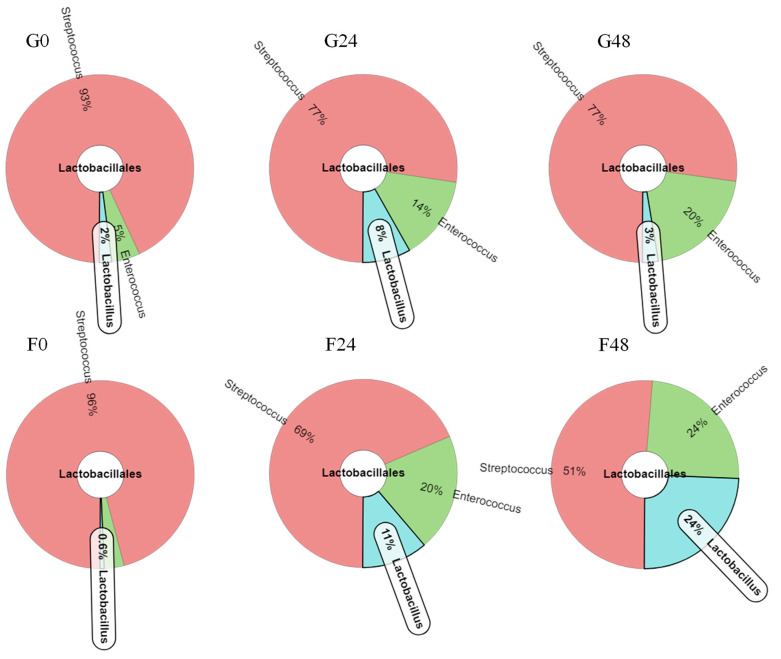
Krona graphs based on relative abundance of bacterial order obtained from the fermentation of glucose (G0, G24, G48) and that of inulin-FOSs extract (F0, F24, F48) at 0, 24 and 48 h of the fermentation processes.

In terms of alpha-diversity, Actinobacteria, Firmicutes and Proteobacteria were the top three phyla obtained from all samples, respectively. These results corresponded to bacteria that had been classified in the families Bifidobacteriaceae, Veillonellaceae and Enterobacteriaceae, which could then be further assigned in the genera *Bifidobacterium*, *Veillonella* and *Klebsiella*, respectively. *Bifidobacterium breve* and *Bb. bifidum* were the two main lactic acid bacteria of the family Bifidobacteriaeae that were obtained from the fermentation process. Moreover, *Veillonella ratti* and *Klebsiella pneumoniae* were recognized as two species belonging to the family Veillonellaceae, respectively. The dynamics of bacterial change during the inulin-FOSs fermentation process were similar to those of the glucose system, but a greater degree of selective stimulation of lactic acid bacteria was observed when compared with glucose (Figure 6). The fecal slurry obtained from a healthy infant showed a broader variety of bacteria than the other samples. The slurry was comprised of 31.1%, 38.4–50.2% and 17.3–25.8% in terms of relative abundance in Phyla Actinobacteria, Firmicutes and Proteobacteria, respectively, while the latter 1.4–4.7% of relative abundance was classified in Phylum Cyanobacteria, Bacteroidetes, Spirochaetes, Gemmatimonadetes, Aminicenantes and Nirospirae. These phyla almost disappeared after 6 h of fermentation in both the inulin-FOSs and glucose-containing media as they were replaced by the phyla Actinobacteria, Firmicutes and Proteobacteria. In turn, these were observed to be the predominant phyla that were involved with certain changes in the bacterial population. It was determined that the classification of the bacteria corresponded to family, genus and species. After 6 h of the fermentation process, inulin-FOS greatly stimulated the growth of *Bb. breve* rather than *Bb. bifidum* due to an increase in relative abundance from 11.3% at the initial stage of fermentation to 55.8% and 74.5% at 6 h and 48 h of the fermentation process, respectively. Under the same conditions, *Bb. bifidum* retained its relative abundance between 15.5 and 24.3% throughout the fermentation period. *V. ratti* was significantly reduced from 45.0% to 0.3% relative abundance. Interestingly, although *K. pneumoniae* was initially found at 1.0% relative abundance, its value was remarkably reduced to 0.1% at the end of the fermentation process. This phenomenon was also detectable in the case of *E. coli* and *St. epidermidis* (data not shown). During glucose fermentation, the profile of bacterial changes was similar to that which was obtained from inulin-FOSs fermentation, but it showed less selectivity in simulating *Bifidobacterium* and less inhibitory effect against *Klebsiella* (Figure 6 and Figure 7). Distinctive differences between fermentation yields of inulin-FOSs and glucose were found when considering the family lactobacillaceae. Inulin-FOSs selectively promoted the growth of *Lactobacillus* and *Streptococcus*, while glucose was determined to be a less specific substrate (Figure 8).

**Figure 9:**
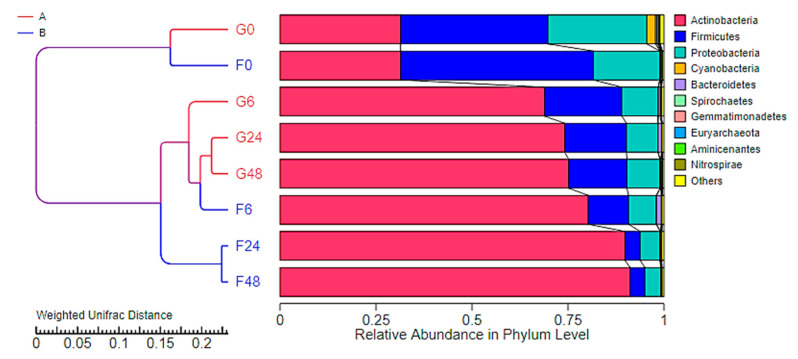
UPGMA cluster tree based on weight unifract distance, revealing the relative abundance in the phylum for the fermentation of inulin-FOSs extract and that of glucose.

In terms of beta-diversity, phylogenetic analysis of the eight samples obtained from the fermentation of inulin-FOSs and glucose supported the substrate specificity of inulin-FOSs towards bacterial diversity. The fermentation of glucose revealed a similar phylogenetic relation to the 6 h fermentation period of the fecal slurry in inulin-FOSs. Therefore, inulin-FOSs could be used as a selective prebiotic substance that would become active within a 6 h period (Figure 9).

### 2.5. Identification of Unknown Sugar

**Figure 10:**
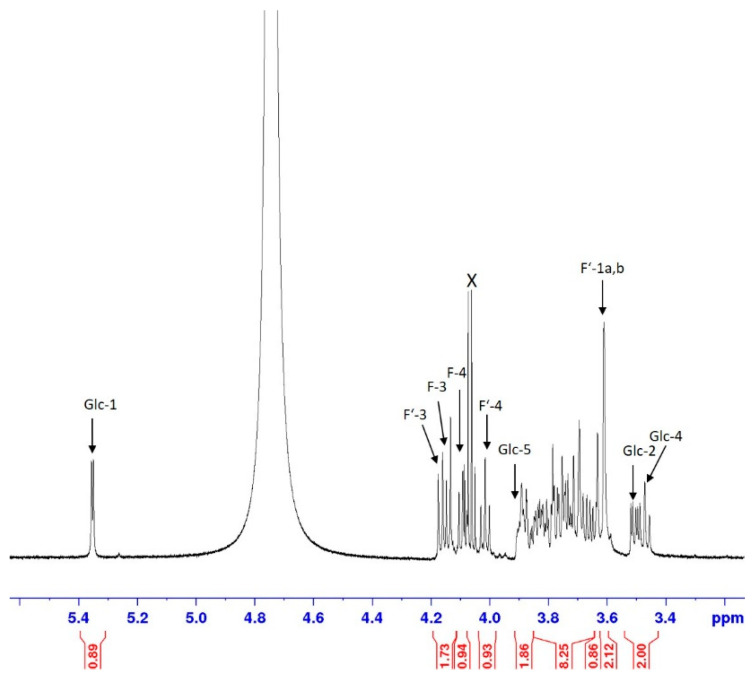
Expansion plot of the 600 MHz ^1^H NMR spectrum. X denotes a contaminant.

The 600 MHz ^1^H NMR spectrum that was recorded in D_2_O showed a low-field shifted anomeric signal at 5.35 ppm, with the value of the homonuclear coupling constant being indicative of an α-linked pyranoside (*J*_1,2_ 3.8 Hz). COSY and TOCSY correlations of the anomeric proton allowed us to identify the complete spin-system. Moreover, together with the *trans*-diaxial coupling constants seen for the H-2 and H-4 signals, the configuration of α-glucopyranoside was confirmed (Figure 10).

**Figure 11:**
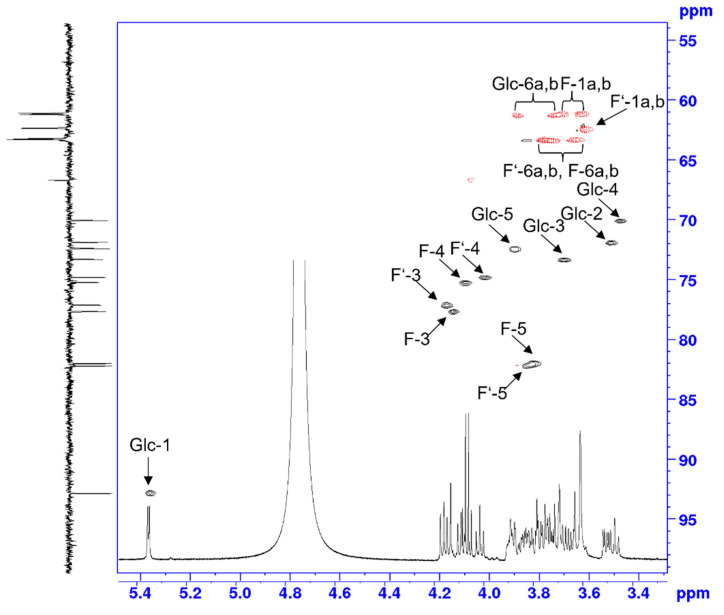
Expansion plot of the gHSQC spectrum of neokestose with ^1^H and ^13^C projections and assigned ^1^H/^13^C NMR signal.

Signals of two fructofuranosyl units were resolved for the doublets of H-3 and the triplets of H-4 within the range of 4.17–4.09 ppm, whereas overlapping bulk signals were observed between 3.90 to 3.61 ppm. The assignment of these signals was then based on the COSY, TOCSY, gHSQC (Figure 11) and HMBC experiments. The discrimination of the two β-linked fructofuranosyl units could not be directly accomplished as the spectral resolution was not sufficient to differentiate their anomeric ^13^C NMR signals at 104.51 ppm. The connectivity between the three saccharide units, however, could be inferred from an HMBC correlation of the separated H-5 signal of the glucose moiety to an anomeric ^13^C NMR signal at 104.51 ppm, which could then be employed to identify the trisaccharide as neokestose. 

An HMBC signal of the anomeric proton of glucose to the ketosidic carbon of a fructofuranoside could not be observed. NOESY correlations from the anomeric proton, however, were present for the C-1 methylene protons at 3.61 ppm, thereby completing the assignment of the sucrose subunit. These assignments (Table S1) were in good agreement with previously published data [14].

## 3. Discussion

The inulin-FOSs extract contained total FOS contents at similar amounts to those reported from *A. cepa* L. [6,15]. White and yellow onions possessed higher RS; thus, they possessed lower DP values than red onions and were not determined to be applicable as a prebiotic. The DP of the red onion was recorded at approximately 5.1 ± 1.1 [16], which was similar to the findings of this study. According to the definition of a prebiotic, any substance to be used as a prebiotic should have at least three criteria including non-digestibility under GIT conditions, fermentability and the selective stimulation of probiotic microorganisms [17]. The inulin-FOSs extract resisted salivary α-amylase, acidity, bile salts and pancreatin, which are key factors of the oral, gastric and small intestinal conditions, respectively. Although the decomposition of inulin-FOSs was significantly found at a pH value of 1.0 for acid hydrolysis, it is generally known that pH values range from 1.9 to 2.5 in the human stomach and can increase to as high as 4.0 to 5.0 depending on the level of fullness in the stomach and the amount of food consumed [18]. It was further revealed that the inulin-FOS extract could enhance the growth of probiotics in a similar manner to glucose, fructose and sucrose, which would imply that the inulin-FOSs acted as simple sugars for probiotics. Both non-neutralized and neutralized fermentation broth of the inulin-FOSs extract exhibited inhibitory effects against not only Gram-positive but also Gram-negative foodborne pathogenic bacteria. In addition, the culture-dependent technique revealed the effect of inulin-FOSs extract on promoting the growth of *Lp. plantarum* TISTR 1500, while displaying an inhibitory effect against *E. coli*, *S. enterica* ser. Thyphimurium and *St. aureus* (data not shown). SCFAs, lactic acid [19] and antimicrobial peptides [20] are common mechanisms of probiotics that act in defense against a potential invasion of enteric pathogens. Furthermore, the inulin-FOSs extract showed a greater bifidogenic effect than glucose. Notably, neokestose exhibited greater potential to be utilized by the mixed culture than 1-kestose and other longer-chain inulin-FOSs. Although neokestose was first observed several decades ago, its availability is limited since it is not generally found in the main sources of commercial FOSs such as chicory and the Jerusalem artichoke. Moreover, it has been rarely reported in microbial production. To date, only onions and asparagus [21] were confirmed as sources of neokestose, as well as agave. However, a recently reported source [22] has currently become commercially unavailable. The inulin-FOSs extract, specifically 1-kestose and neokestose, showed a significant level of potential to elevate the relative abundance of *Bifidobacterium*, specifically *Bb. breve*, and to diminish the amounts of *Klebsiella* and *Escherichia-Shigella*, as has been described in previous report [23]. However, the constant relative abundance of *Bb. bifidum* was retained. Both *Bb. breve* and *Bb. bifidum* present in the batch cultivation were determined to be related to infant-type *Bifidobacterium,* in agreement with its origin. Remarkably, *Bb. bifidum* had no β-fructofuranosidase-encoding gene, and thus it lacked the ability to utilize FOSs, particularly neokestose and 1-kestose [24]. On the other hand, *Bb. breve* exhibited even greater merit due to the presence of four type-gene clusters associated with FOSs degradation, including β-fructofuranosidase-encoding gene [25]. Thus, it can be implied that *Bb. breve* is the key microorganism of utilization of neokestose and 1-kestose. These results were relevant to evidence found in *Bb. longum*, another infant-type Bifidobacterium [26]. However, the promotion of *Bb. breve* growth in this study was likely due to neokestose utilization. An increase in the relative abundance of *Bb. bifidum* may have been caused by the glucose, fructose and sucrose present in the inulin-FOSs extract. The inulin-FOSs extract also selectively promoted the relative abundance of *Lactobacillus* and *Enterococcus,* in agreement with its selective fermentation by representative probiotic lactic acid bacteria. To the best of our knowledge, there has been no report involving a comparison of the bifidogenic effects of 1-kestose and neokestose. Here, it is likely to be concluded that neokestose displayed a stronger vital role than 1-kestose on the growth of *Bb. breve*. A recent study revealed that *Bb. breve* preferentially fermented 1-kestose and minimally fermented nystose in FOSs, which was relevant to the results of this study [27]. Profiles of SCFAs in this study were similar to those of the 1-kestose [27] and neokestose [23] fermentation processes by *Bb. breve*, in which lactic acid and acetic acid were determined to be the main organic acids. Beta-diversity indicated that inulin-FOSs could more selectively establish good bacterial microbiota than glucose. Red onion syrup is generally a homemade product that has been used as a natural home remedy for coughs. It is believed that the strong vapors of the syrup can help to stop coughing [28]. In addition, it can be developed into a ready-to-eat product in the form of a salad dressing. Other functional benefits of the red onion are its flavonoids and pigment, which are mainly quercetin glycosides [29] and anthocyanin [30], respectively. Additionally, neokestose has acted as an additional or chemopreventive therapeutic agent in the treatment of melanoma [31]. With regard to the remaining oligosaccharides in the fermentation process, it can be suggested that inulin-FOSs extract can be used in the form of a functional syrup and a food additive and may be feasible for pharmaceutical application.

## 4. Materials and Methods

### 4.1. Chemicals

All chemicals used for the analysis of simulated salivary fluids, simulated gastric fluids and simulated intestinal fluids were of analytical grade and were made available from RCI Labscan (Bangkok, Thailand). Salivary α-amylase and pancreatin were purchased from Sigma-Aldrich (St. Louis, MO, USA). Bile salts, hemin, vitamin K1, cysteine HCl and the appropriate media were purchased from HiMedia (Nashik, India). Standard short chain fatty acids (SCFA), including propionic acid, butyric acid, acetic acid and lactic acid, were of HPLC grade and were obtained from RCI Labscan.

### 4.2. Microorganisms and Culture Conditions

Probiotic bacteria, including *Lc. casei* TISTR 1500, *Lc. casei* TISTR 1463, *Lp. plantarum* TISTR 1465 and *Lp*. *plantarum* TISTR 2075, were obtained from Thailand Institute of Scientific and Technological Research (TISTR). They were maintained in deMan Rogosa and Sharpe (MRS) broth containing 15% (*v*/*v*) glycerol and stored at −80 °C. When necessary, each bacterium was static-cultured in MRS broth at 37 °C for 24 h and then plated on MRS agar using the streak plate technique. Bacterial pathogens, including *B. cereus* TISTR 747, *E. coli* TISTR 527, *St. aureus* TISTR 746 and *S. enterica* ser. Thyphimurium TISTR 1472, were maintained in nutrient broth (NB) while *L. monocytogenes* DMST 17303 was maintained in trypticase soy broth (TSB). The first four bacteria were grown aerobically on nutrient agar (NA) at 37 °C. On the other hand, *L. monocytogenes* was grown anaerobically on trypticase soy agar (TSA) in an anaerobic jar at 37 °C.

### 4.3. Extraction and Standardization of Inulin-FOSs Extract

Red onions (*A. cepa* var. *viviparum*) were purchased from the Muang Mai Market (located at 18.797334, 98.9951373). The extraction of inulin-FOSs was conducted according to the method previously described [8]. Briefly, 1 kg of red onions were chopped into small cubes (1 × 1 × 1 cm) for further homogenization and extraction using a JT-2010 Healthy Slow Juicer (Jutian, China). The onion extract was heated to 80 °C for 30 min, left to cool and centrifuged at 40,000× *g* for 10 min in order to remove the insoluble fraction. The clear supernatant was assigned as red onion extract and was determined for total carbohydrate content using the phenol–sulfuric acid method [32]. Reducing sugars were then determined by the 3,5-dinitrosalicylic acid (DNS) method [33], while sugar residues, such as glucose, fructose and sucrose, were determined by high-performance liquid chromatography (HPLC).

### 4.4. Simulated Oral Conditions

Inulin-FOSs extract (50 μL at an initial concentration of 100 mg/mL) was mixed with 375 μL of simulated salivary fluid (SSF) (15.1 mM KCl, 3.7 mM KH_2_PO_4_, 13.6 mM NaHCO_3_, 0.15 mM MgCl_2_·6H_2_O, 0.06 mM (NH_4_)_2_CO_3_, 1.5 mM CaCl_2_·2H_2_O, pH 7.0), 25 μL of 80 U/mL salivary α-amylase dissolved in SSF, 1.25 μL of 0.3 M CaCl_2_ and 48.75 μL of water. The reaction mixture was incubated at 37 °C for 120 min. Samples were periodically taken at 30 min intervals. The samples were then placed in a 100 °C dry bath for 10 min to terminate the reaction of the enzymes prior to the quantitative analysis of reducing sugars using the DNS method and qualitative analysis by thin layer chromatography (TLC).

### 4.5. Simulated Gastric Conditions

Inulin-FOSs extract at an initial concentration of 10 g/L total carbohydrate was adjusted to pH 2.0 and pH 3.0 with 0.2 M HCl and was subsequently incubated at 37 °C. After being incubated for 0, 30, 60, 90 and 120 min, samples were taken and neutralized with 0.2 M NaHCO_3_ for further quantitative analysis of reducing sugars by applying the DNS method and qualitative analysis by TLC.

### 4.6. Simulated Pancreatic Conditions

Inulin-FOSs extract at an initial concentration of 100 mg/mL (50 μL) was mixed with 375 μL of simulated intestinal fluid (SIF) (6.8 mM KCl, 0.8 mM KH_2_PO_4_, 85 mM NaHCO_3_, 38.4 mM NaCl, 0.33 mM MgCl_2_, 0.6 mM CaCl_2_ and 25 μL of 800 U/mL pancreatin solution), 31.25 μL of SSF, 1.25 μL of 0.3 M CaCl_2_ and 48.75 μL of water. The reaction mixture was incubated at 37 °C for 120 min. The samples were then placed in a 100 °C dry bath for 10 min to terminate the reaction of the enzymes prior to quantitative analysis of reducing sugars using DNS method and qualitative analysis by TLC.

### 4.7. Fermentation of Inulin-FOSs by Various Probiotic Bacteria and Inhibitory Effect of Fermentation Broth

The fermentability of inulin-FOSs extract was determined using different probiotic strains including *Lc. casei* TISTR 1500, *Lc. casei* TISTR 1463, *Lp. plantarum* TISTR 1465 and *Lp*. *plantarum* TISTR 2075. Modified MRS broth (10 g/L peptone, 10 g/L beef extract, 5 g/L yeast extract, 1 g/L tween80, 2 g/L K_2_HPO_4_, 5 g/L CH_3_COONa·3H_2_O, 2 g/L tri-ammonium hydrogen citrate, 0.2 g/L MgSO_4_·7H_2_O, 0.2 g/L MnSO_4_·H_2_O, pH 6.8) was prepared using 10 g/L of total carbohydrate derived from inulin-FOSs as the sole carbon source. A total of 1% (*v*/*v*) of the inoculum of each probiotic strain was transferred to the modified MRS broth and statically incubated at 37 °C for 24 h. Samples were periodically taken every 6 h in order to measure an absorbance at 600 nm. The remaining samples were then centrifuged at 17,350× *g* and at 4 °C for 10 min. The clear supernatant was used for the qualitative analysis of the inulin-FOSs profile by TLC. The process was divided into two parts. In the first part, the fraction was neutralized to pH 6.8 by the addition of 0.1 M NaOH, while the rest was considered a non-neutralized fraction. Both fractions were sterilized by being passed through a sterile membrane filter with a pore size of 0.22 μm. For antimicrobial activity assay, different foodborne pathogens, including *B. cereus*, *E. coli*, *S. enterica* ser. Thyphimurium, *St. aureus* and *L. monocytogenes*, were separately spread on the appropriate medium plate. The plates were punched with a 0.6 mm diameter corkborer to make the agar wells. The filtered sterile fraction (50 μL) was then applied on the agar well. Plates were incubated aerobically at 37 °C for the cultivation of *B. cereus*, *E. coli*, *S. enterica* ser. Thyphimurium and *St. aureus*, but were separately incubated in an anaerobic jar with a Gas-Pak at the same incubation temperature for *L. monocytogenes*. After 24 h of incubation, the inhibition zone around the agar well was measured and recorded. 

### 4.8. In Vitro Batch Culture Fermentation

A fresh fecal sample was collected from a healthy infant who had no record of receiving antibiotics, prebiotics or probiotics since birth and for whom there was no recent evidence of any gastrointestinal disorders. Within 2 h of the initial stool collection, the fresh fecal sample was diluted with PBS to produce a final concentration of 10% (*v*/*v*). Then, 1% (*v*/*v*) inoculum was transferred into a laboratory bottle containing 100 mL of the basal medium (10 g/L inulin-FOSs extract, 2 g/L peptone, 2 g/L yeast extract, 0.1 g/L NaCl, K_2_HPO_4_ 0.04 g/L, 0.04 g/L KH_2_PO_4_, 0.01 g/L MgSO_4_·7H_2_O, 0.01 g/L CaCl_2_·7H_2_O, 2 g/L tween80, 0.02 g/L hemin, 10 mL/L vitamin K1, 0.5 g/L cysteine HCl and 0.5 g/L bile salts, pH 7). After adding the inoculum, sterile N_2_ was added to the laboratory bottle. The bottle was then placed on a 50-rpm rotary shaker and maintained at 37 °C. Samples were collected at 0, 12, 24 and 48 h of the fermentation process by centrifugation at 10,380× *g* at 4 °C for 15 min. The obtained pellet was used for further DNA extraction, while the supernatant was used for the determination of total carbohydrate, glucose, fructose and sucrose contents. The relevant pH value, profile of the inulin-FOSs and quantity of the SCFAs were also determined.

### 4.9. Extraction of Genomic DNA and Amplicon Sequencing of 16S rRNA Gene

Genomic DNA was extracted using a Wizard^®^ Genomic DNA purification kit (Promega Corp., Madison, WI, USA) according to the manufacturer’s instructions. The DNA quality and quantity were assessed by ratios of 260/280 nm and 260/230 nm. Genomic DNA was used as a template for PCR amplification of bacterial 16S rRNA gene [34]. For amplicon sequencing, the V3–V4 region of the 16S rRNA gene was amplified using the previously described protocol [35]. Briefly, high-throughput sequencing was conducted from the sequenced libraries of bacterial 16S rRNA genes using TruSeq^®^ DNA PCR-Free Sample Preparation Kit (Illumina, San Diego, CA, USA) and index codes were added. The Qubit@ 2.0 Fluorometer (Invitrogen, Thermo Scientific, CA, USA) and Agilent Bioanalyzer 2100 system were used to assess library quality. The library was sequenced on an Illumina HiSeq2500 platform (Illumina, San Diego, CA, USA) by Novogene Bioinformatics Technology Co., Ltd. (Beijing, China).

### 4.10. Bioinformatics

FLASH software version 1.2.7 [36] was used to merge results, while QIIME software version 1.7 [37] was used to filter the raw sequencing reads obtained from Illumina platform prior to applying standards of quality control. The mature sequencing reads were clustered into operation taxonomic units (OTUs) using UPARSE software version 7.0 [38], for which the threshold was set at 97% sequence similarity. A standard sequence number that corresponded to the sample with the least sequences was used to normalize the OTUs abundance number. Unweighted Pair-Group Method with Arithmetic Means (UPGMA) clustering was performed as a type of hierarchical clustering method to interpret the distance matrix using average linkage and was also conducted by QIIME software.

### 4.11. Thin Layer Chromatography and Isolation of Unknown Sugar

A sample of 1 μL was spotted on an aluminum silica gel plate. After drying with a dryer, it was developed in a chamber containing *n*-butanol/ethanol/water (5:3:2 *v*/*v*/*v*). The developing procedure was conducted three times to separate monosaccharides and FOSs better. Then, the TLC plate was dried and sprayed with 0.5% (*w*/*v*) thymol in a mixed solution of 5% (*v*/*v*) H_2_SO_4_ in ethanol prior to heating at 100 °C for 10 min to visualize monosaccharides and FOSs in pink color. For the isolation of unknown sugar, a glass silica gel plate was used as a stationary phase. A sample of 10 μL was spotted on the gel plate, and the same procedure was performed as above. The spot of unknown sugar was collected by scraping the silica gel plate and was then dissolved with deionized water and centrifuged at 17,350× *g* for 10 min. The supernatant was filtered through a 0.22 μm filter cartridge and lyophilized to a powder.

### 4.12. Structural Identification of Unknown Sugar by Nuclear Magnetic Resonance

NMR spectra were recorded on a Bruker AV III 600 MHz NMR spectrometer (Bruker BioSpin, Rheinstetten, Germany). The software version and all pulse and parameter sets were based on Topspin 3.5 PL 6. Furthermore, spectra were acquired at 27 °C in D_2_O as the solvent and referenced externally for ^1^H to DSS in D_2_O (δ = 0 ppm) and for ^13^C to dioxane in D_2_O (67.4 ppm). For all 1D and 2D NMR experiments, the appropriate pulse sequences were used in the same manner as they had been supplied by the manufacturer. The structure was elucidated based on COSY, NOESY, TOCSY, HSQC and HMBC correlation spectra. HMBC spectra were recorded with a resolution of F2 (4096), F1 (1024) resulting in a FIDRES F1 of 65.5 Hz. Non-uniform sampling was used with a NUS amount of 25%, resulting in 256 NUS points. HSQC spectra were recorded with a resolution of F2 (1024) and F1 (1024), resulting in a FIDRES F1 of 61.9 Hz. Furthermore, non-uniform sampling was used with a NUS amount of 25%, resulting in 128 NUS points. All other 2D experiments were performed in the traditional (planes) acquisition mode. For TOCSY, a mixing time of 80 ms was used, while a mixing time of 300 ms was employed for NOESY. 

### 4.13. Analytical Methods

Total carbohydrates were determined using the phenol–sulfuric acid method [32]. Briefly, 0.25 mL of sample was mixed with 0.25 mL of 5% (*w*/*v*) phenol. Then, 1.25 mL of sulfuric acid was added and thoroughly mixed. The reaction was carried out at room temperature (25 °C) for 30 min. Finally, an absorbance at 490 nm was measured. Glucose was used as the standard.

Total reducing sugars were determined using the DNS method [33]. Briefly, 0.1 mL of sample was mixed with 0.1 mL of DNS solution. Then, the reaction was carried out at 100 °C for 10 min. After that, 0.8 mL of distilled water added prior to measuring an absorbance at 540 nm. Glucose was used as the standard.

Glucose, fructose and sucrose were analyzed by high-performance liquid chromatography (HPLC) using an Asahipak (NH_2_P-50 4E) column equilibrated with a solution of acetonitrile/H_2_O (75:25 *v*/*v*). The conditions were carried out at 30 °C with a flow rate of 0.6 mL/min. The separated sugars were detected by refractive index (RI) detector. 

SCFAs, including acetic acid, lactic acid, propionic acid and butyric acid, were analyzed by HPLC according to the previously described method [39].

All experiments were performed in duplicate. The results were presented as mean ± standard deviation. Data analysis of the mean values was performed based on a full-factorial complete randomized design (CRD). Briefly, data were subjected to analysis of variance (ANOVA), and multiple comparison tests were performed based on all paired comparisons, using Tukey’s HSD test at the 95% confidence level. All analyses were carried out using the Statistix software version 8.0 (Analytical Software, Tallahassee, FL, USA). A probability value of *p* < 0.05 was considered significant.

## 5. Conclusions

This paper presents the first finding showing that neokestose from red onion could selectively enhance bifidogenic effects more effectively than 1-kestose and documents the alternative merits of red onion extract, verifiying that it is applicable to be used in the food and pharmaceutical industries. For further study, it is important to prepare short-chain inulin-type fructan and inulin-type neofructan in addition to removing the residual sugar, namely glucose, fructose and sucrose, in order to extensively encourage more prebiotic effects. 

## Figures and Tables

**Table 1:** Summary of inulin-FOSs extract obtained from red onion.

Red Onion (kg)	Extract Volume (L)	Total Carbohydrate (g/L)	FGS ^1^ (g/L)	Total Inulin-FOSs ^2^ (g/L)	Average DP ^3^	Inulin-FOSs Yield ^4^ (g/100 g)
20.0	10.0	98.00 ± 2.8	24.0 ± 0.38	74.0 ± 0.47	4.1 ± 0.13	3.7 ± 0.14

^1^ FGS was the sum of the sugars fructose (F), glucose (G) and sucrose (S); ^2^ Total inulin-FOSs were calculated by subtracting of FGS from total carbohydrate; ^3^ DP was calculated by total carbohydrate divided by FGS; ^4^ Inulin-FOSs yield was calculated based on total inulin-FOSs obtained from 100 g red onions.

**Table 2:** Inhibitory effect of inulin-FOSs fermentation broth against foodborne pathogenic bacteria.

Probiotic Bacteria	Fermentation Broth	*E. coli*	*B. cereus*	*S. enterotica* ser. Thyphimurium	*St. aureus*	*L. monocytogenes*
1463	Non-neutralized	0.75 ± 0.07 ^a^	1.15 ± 0.07 ^a^	0.95 ± 0.07	0.95 ± 0.07	0.90 ± 0.00 ^a^
	Neutralized	0.25 ± 0.07 ^b^	0.25 ± 0.07 ^b^	0.6 ± 0.14	0.70 ± 0.14	0.20 ± 0.00 ^b^
1465	Non-neutralized	0.75 ± 0.07	0.60 ± 0.14	0.65 ± 0.07 ^a^	1.15 ± 0.07 ^a^	0.90 ± 0.00 ^a^
	Neutralized	0.60 ± 0.00	0.45 ± 0.07	0.40 ± 0.00 ^b^	0.50 ± 0.00 ^b^	0.15 ± 0.07 ^b^
1500	Non-neutralized	0.75 ± 0.07 ^a^	1.15 ± 0.21 ^a^	0.90 ± 0.00	1.00 ± 0.00 ^a^	0.85 ± 0.07 ^a^
	Neutralized	0.35 ± 0.07 ^b^	0.45 ± 0.07 ^b^	0.70 ± 0.14	0.55 ± 0.07 ^b^	0.10 ± 0.00 ^b^

^a,b^ refer to significant difference of inhibition zones of non-neutralized and neutralized fermentation broths of each probiotic bacterium.

## Data Availability

The data presented in this study are available in the article and Supplementary Material.

## Supplementary Materials

The following are available online at https://www.mdpi.com/article/10.3390/plants10112401/s1, Table S1: ^1^H and ^13^C NMR chemical shifts and homonuclear coupling constants of neokestose.

## References

[1] Fujishima M., Furuyama K., Ishihiro Y., Onodera S., Fukushi E., Benkeblia N., Shiomi N. (2009). Isolation and structural analysis in vivo of newly synthesized fructooligosaccharides in onion bulbs tissues (*Allium cepa* L.) during storage. Int. J. Carbohydr. Chem..

[2] Kumar C.G., Sripada S., Poornachandra Y., Grumezescu A.M., Holban A.M. (2018). Chapter 14-Status and future prospects of fructooligosaccharides as nutraceuticals. Role of Materials Science in Food Bioengineering.

[3] Shiomi N., Benkeblia N., Onodera S. (2005). The Metabolism of the Fructooligosaccharides in Onion Bulbs: A Comprehensive Review. J. Appl. Glycosci..

[4] Rastall R.A. (2010). Functional oligosaccharides: Application and manufacture. Annu. Rev. Food Sci. Technol..

[5] Kwak J.-H., Seo J.M., Kim N.-H., Arasu M.V., Kim S., Yoon M.K., Kim S.-J. (2017). Variation of quercetin glycoside derivatives in three onion (*Allium cepa* L.) varieties. Saudi J. Biol. Sci..

[6] Jaime L., Martín-Cabrejas M.A., Mollá E., López-Andréu F.J., Esteban R.M. (2001). Effect of storage on fructan and fructooligosaccharide of onion (*Allium cepa* L.). J. Agric. Food Chem..

[7] Wongputtisin P. (2003). Selection of Oligosaccharides from Some Local Plants for Utilizing as Prebiotics. Master’s Thesis.

[8] Nanti S., Wongputtisin P., Chomsri N.-o., Deejing S., Niamsup P. (2016). Primary prebiotic properties of Thai white sausage (Moo-yor) supplemented with fructooligosaccharides extracted from onion (*Allium cepa* L.) and chicory root. J. Agric. Sci..

[9] Gibson G.R., Hutkins R., Sanders M.E., Prescott S.L., Reimer R.A., Salminen S.J., Scott K., Stanton C., Swanson K.S., Cani P.D. (2017). Expert consensus document: The International Scientific Association for Probiotics and Prebiotics (ISAPP) consensus statement on the definition and scope of prebiotics. Nat. Rev. Gastroenterol. Hepatol..

[10] Spacova I., Dodiya H.B., Happel A.-U., Strain C., Vandenheuvel D., Wang X., Reid G. (2020). Future of probiotics and prebiotics and the implications for early career researchers. Front. Microbiol..

[11] Samanwong T., Ruangdechboonyarit N., Muangham S., Aeamsri S. (2016). Survival of encasulated probiotics bacteria in yoghurt from corn milk, cow milk and soy milk as preserved at low temperature. Huachiew Chalermprakiet Sci. Technol. J..

[12] Chaipojjana R. (2014). Production of Bioactive Powder by Double Emulsion. Master’s Thesis.

[13] Lapsiri W., Nitisinprasert S., Wanchaitanawong P. (2011). *Lactobacillus plantarum* strains from fermented vegetables as potential probiotics. Kasetsart J. Nat. Sci..

[14] Liu J., Waterhouse A.L., Chatterton N.J. (1991). Proton and carbon chemical-shift assignments for 6-kestose and neokestose from two-dimensional n.m.r. measurements. Carbohydr. Res..

[15] Rodríguez Galdón B., Tascón Rodríguez C., Rodríguez Rodríguez E.M., Díaz Romero C. (2009). Fructans and major compounds in onion cultivars (*Allium cepa*). J. Food Compost. Anal..

[16] Jurgiel-Malecka G., Gibszynska M., Nawrocka-Pezik M. (2015). Comparison of chemical composition of selected cultivars of white, yellow and red onions. Bulg. J. Agric. Sci..

[17] Roberfroid M. (2007). Prebiotics: The concept revisited. J. Nutr..

[18] Minekus M., Alminger M., Alvito P., Ballance S., Bohn T., Bourlieu C., Carrière F., Boutrou R., Corredig M., Dupont D. (2014). A standardised static in vitro digestion method suitable for food—An international consensus. Food Funct..

[19] Lamas A., Regal P., Vázquez B., Cepeda A., Franco C.M. (2019). Short chain fatty acids commonly produced by gut microbiota influence *Salmonella enterica* motility, biofilm formation, and gene expression. Antibiotics.

[20] Sun Y., O’Riordan M.X.D. (2013). Regulation of bacterial pathogenesis by intestinal short-chain fatty acids. Adv. Appl. Microbiol..

[21] Mitsuoka T., Hidaka H., Eida T. (1987). Effect of fructo-oligosaccharides on intestinal microflora. Nahrung.

[22] García-Curbelo Y., López M.G., Bocourt R., Collado E., Albelo N., Nuñez O. (2016). Structural characterization of fructans from Agave fourcroydes (Lem.) with potential as prebiotic. Cuba. J. Agric. Sci..

[23] Kilian S., Kritzinger S., Rycroft C., Gibson G., du Preez J. (2002). The effects of the novel bifidogenic trisaccharide, neokestose, on the human colonic microbiota. World J. Microbiol. Biotechnol..

[24] Omori T., Ueno K., Muramatsu K., Kikuchi M., Onodera S., Shiomi N. (2010). Characterization of recombinant β-fructofuranosidase from *Bifidobacterium adolescentis* G1. Chem. Cent. J..

[25] Liu S., Fang Z., Wang H., Zhai Q., Hang F., Zhao J., Zhang H., Lu W., Chen W. (2021). Gene–Phenotype associations involving human-residential bifidobacteria (HRB) reveal significant species- and strain-specificity in carbohydrate catabolism. Microorganisms.

[26] Omori T., Ueno K., Kikuchi M., Onodera S., Shiomi N. (2010). Properties of recombinant and β-fructofuranosidase from *Bifidobacterium longum* JCM1217. J. Appl. Glycosci..

[27] Tochio T., Kadota Y., Tanaka T., Koga Y. (2018). 1-kestose, the smallest fructooligosaccharide component, which efficiently stimulates *Faecalibacterium prausnitzii* as well as *Bifidobacteria* in humans. Foods.

[28] Sultana S., Khan A., Safhi M.M., Alhazmi H.A. (2016). Cough suppressant herbal drugs: A review. Int. J. Pharm. Sci. Invent..

[29] Grzelak K., Milala J., Król B., Adamicki F., Badełek E. (2009). Content of quercetin glycosides and fructooligosaccharides in onion stored in a cold room. Eur. Food Res. Technol..

[30] Ferreres F., Gil M.I., Tomás-Barberán F.A. (1996). Anthocyanins and flavonoids from shredded red onion and changes during storage in perforated films. Int. Food Res. J..

[31] Wu J.S., Chang J.Y., Chen C.W., Lin M.T., Sheu D.C., Lee S.M. (2017). Neokestose suppresses the growth of human melanoma A2058 cells via inhibition of the nuclear factor-κB signaling pathway. Mol. Med. Rep..

[32] DuBois M., Gilles K.A., Hamilton J.K., Rebers P.A., Smith F. (1956). Colorimetric method for determination of sugars and related substances. Anal. Chem..

[33] Miller G.L. (1959). Use of dinitrosalicylic acid reagent for determination of reducing sugar. Anal. Chem..

[34] Kanpiengjai A., Mahawan R., Lumyong S., Khanongnuch C. (2016). A soil bacterium *Rhizobium borbori* and its potential for citrinin-degrading application. Ann. Microbiol..

[35] Unban K., Khatthongngam N., Pattananandecha T., Saenjum C., Shetty K., Khanongnuch C. (2020). Microbial community dynamics during the non-filamentous fungi growth-based fermentation process of Miang, a traditional fermented tea of north Thailand and their product characterizations. Front. Microbiol..

[36] Magoč T., Salzberg S.L. (2011). FLASH: Fast length adjustment of short reads to improve genome assemblies. Bioinformatics.

[37] Caporaso J.G., Kuczynski J., Stombaugh J., Bittinger K., Bushman F.D., Costello E.K., Fierer N., Peña A.G., Goodrich J.K., Gordon J.I. (2010). QIIME allows analysis of high-throughput community sequencing data. Nat. Methods.

[38] Edgar R.C. (2013). UPARSE: Highly accurate OTU sequences from microbial amplicon reads. Nat. Methods.

[39] Kanpiengjai A., Lumyong S., Pathom-aree W., Khanongnuch C. (2014). Starchy effluent from rice noodle manufacturing process as feasible substrate for direct lactic acid production by *Lactobacillus plantarum* S21. J. Korean Appl. Biol. Chem..

