# Supplementary material for: Potential of Inulin-Fructooligosaccharides Extract Produced from Red Onion (Allium cepa var. viviparum (Metz) Mansf.) as an Alternative Prebiotic Product
Source: Plants (Basel). 2021 Nov 7;10(11):2401. doi: 10.3390/plants10112401 (PMC8624415; doi:10.3390/plants10112401)
Supplement: Supplementary file 1 [file plants-10-02401-s001.zip › plants-1459996-supplementary.pdf]

Table S1. ^1H and ^{13}C NMR chemical shifts and homonuclear coupling constants of neokestose.

Residue	Atom number	^1H (ppm)	*J* (Hz)	^{13}C (ppm)	HMBC correlations
β-Fru*f*-(2→	1a	3.70		61.04	
	1b	3.63		-	
	2	-		104.51	
	3	4.14	8.5	77.62	
	4	4.09	8.1	75.17	
	5	3.82		81.96	75.2
	6a	3.75		63.24	
	6b	3.66		-	82.0
→6)-α-Glc*p*-(1→	1	5.35	3.8	92.82	104.4,73.2,72.3
	2	3.51	3.8, 10.0	71.82	
	3	3.70	n.d.	73.25	
	4	3.47	9.8, 9.8	70.00	
	5	3.89		72.35	
	6a	3.88		61.14	
	6b	3.74			
→2)-β-Fru*f*	1a	3.61		62.28	104.5, 77.1
	1b	3.61		-	
	2	-		104.51	
	3	4.17	8.7	77.07	62.3
	4	4.015	8.9	74.76	82.1
	5	3.84		82.16	
	6a	3.79		63.16	
	6b	3.74		-	
